# Epidemiology of *Mycoplasma pneumoniae* and co-detection with other respiratory pathogens in a respiratory sentinel surveillance system in Scotland, October 2022 to May 2025

**DOI:** 10.2807/1560-7917.ES.2026.31.29.2500845

**Published:** 2026-07-23

**Authors:** Helen Gadegaard, Mark Hamilton, Catríona Oliver, Pamela Saunders, Rory Gunson, Kimberly Marsh, Michael L Beeton, Josie Evans

**Affiliations:** 1 Public Health Scotland, Glasgow, Scotland; 2 West of Scotland Specialist Virology Service, Glasgow, Scotland; 3 Cardiff School of Sport and Health Sciences, Cardiff Metropolitan University, Cardiff, Wales

**Keywords:** Respiratory infection, Mycoplasma pneumonia, co-infection, respiratory disease, sentinel surveillance

## Abstract

**BACKGROUND:**

Community-acquired pneumonia by *Mycoplasma pneumoniae* is often complicated by co-infections with other respiratory pathogens.

**AIM:**

We describe through a sentinel respiratory surveillance system in Scotland, *M. pneumoniae* infection occurrence in patients presenting to general practitioners with acute respiratory infection (ARI) (October 2022−May 2025) and the co-detection frequency of other respiratory pathogens.

**METHODS:**

Upper respiratory tract swabs from a representative community sample of 65,798 ARI patients were laboratory-tested for 10 respiratory pathogens, including *M. pneumoniae*. Positivity for *M. pneumoniae* was monitored over time. Single or multiple joint pathogen detections were assessed and stratified by demographic characteristics. Proportions hospitalised within 14 days after their *M. pneumoniae* positive test were determined.

**RESULTS:**

Of 65,798 patients (40,158 female, 25,534 male, 106 sex unknown; median age: 35 years, interquartile range (IQR): 17–59), 3,031 (4.6%) were *M. pneumoniae* positive (1,686 female, 1,340 male, five sex unknown; median age: 18 years, IQR: 9–39)*.* Positivity was elevated in October 2023−October 2024, particularly in 5–14-year-olds, and peaked at 47.7% (31/65) in week 1, 2024. Among *M. pneumoniae* cases viably tested for all pathogens, 26.0% (728/2,799) had co-detections of another pathogen, with rates in 0–4-year-olds (61.3%; 122/199) and 5–14-year-olds (31.3%, 321/1,024) reflecting those reported elsewhere in hospitalised paediatric cases. Co-detections involving rhinovirus (47.5%; 346/728) predominated, otherwise varying by pathogen and age. Of 3,031 *M. pneumoniae* cases, 1.8% (n = 55) were admitted.

**CONCLUSION:**

Sentinel surveillance of respiratory pathogens in the community was helpful to characterise an *M. pneumoniae *epidemic, revealing frequent respiratory-pathogen co-detections in ≤ 14-year-olds, as prior reported in hospitalised *M. pneumoniae* paediatric cases.

**Key public health message**

**What did you want to address in this study and why?**

Pneumonia due to *Mycoplasma pneumoniae* infections can be complicated by co-infections with additional respiratory pathogens. Between the autumns of 2023 and 2024, high numbers of patients with *M. pneumoniae* were observed among people with acute respiratory infection attending general practices in Scotland. We sought to describe the demographic affected, and the incidence of other respiratory pathogens co-detected with *M. pneumoniae*.

**What have we learnt from this study?**

Proportions of patients testing positive for *M. pneumoniae* were elevated in 2023/24, notably in school-aged children. Co-detections of *M. pneumoniae* with other respiratory pathogens occurred at higher levels in children than in adults, with 61% of individuals under 4-year-olds co-infected. Rhinovirus was the most commonly co-detected virus. Paediatric co-detection rates in the community reflected those prior reported in severely ill hospitalised children.

**What are the implications of your findings for public health?**

Community testing of respiratory pathogens, which was conducted through a sentinel respiratory surveillance system in Scotland enabled characterisation of an *M. pneumoniae *epidemic and uncovered frequent respiratory-pathogen co-infections in children under 14-year-olds, demonstrating the usefulness of this type of surveillance.

## Introduction

*Mycoplasma pneumoniae* is subject to surveillance worldwide as a common cause of community-acquired pneumonia (CAP) in children [1-5]. A fastidious bacterium lacking a cell wall, *M. pneumoniae* causes atypical pneumonia that can manifest as severe disease particularly in children [6,7] with increased clinical significance often associated with respiratory co-infections [8-10]. Furthermore, serious extrapulmonary complications can occur upon *M. pneumoniae* infection in both children and adults [11-13]. Of concern too is the incidence of macrolide-resistant *M. pneumoniae* currently notable in Asia, and the consequences of treatment failure due to antibiotic resistance [14,15].

In terms of its epidemiology,* M. pneumoniae* has previously been characterised as causing cyclical seasonal epidemic episodes, with a pattern of 1–3 year-recurrence of epidemics in various geographic locations [16] and a mean epidemic duration of 18 months in England and Wales [17]. Since the COVID-19 pandemic, these patterns have been disrupted [4,18]. Re-emergence of *M. pneumoniae* post-pandemic has appeared delayed when compared to other respiratory pathogens, as non-pharmaceutical interventions against COVID-19 were eased and lifted globally [19-23].

Several clinical studies have previously indicated the prevalence of co-infecting respiratory viruses in children hospitalised with *M. pneumoniae* pneumonia (MPP), with co-detection shown to be associated with severity of disease, drug resistance and requirement for additional therapy [10]. Of 107 paediatric MPP cases admitted to a children’s hospital in Shanghai between December 2016 and May 2019, the co-infection rate was found to be 56%, with adenovirus identified as the most prevalent co-infecting organism, detected in 22% of cases [24]. Adenovirus was similarly found to be the most common pathogen to co-infect with *M. pneumoniae* in a 2019 study of paediatric severe CAP in another Chinese hospital [25] and these two pathogens were identified as a risk factor for severe CAP in children [26]. In a Russian study of paediatric MPP admission at a children’s hospital between October 2023 and February 2024, 62% (119/193) of cases had at least one co-infecting virus detected, with human parainfluenza viruses being the most frequent [27]*. *Co-infections in *M. pneumoniae* cases of hospitalised paediatric pneumonia but detected with more common seasonal respiratory viruses, such as influenza virus (in a 2010*–*2016 study) [9] and rhinovirus have been reported in studies from 2017 [28], 2016*–*2019 [24] and 2019*–*2020 [8]. Less is known about the prevalence of co-infection of *M. pneumoniae* outside hospital settings. Respiratory sentinel surveillance programmes in primary care settings often provide detection rates of individual pathogens without the simultaneous detection of multiple pathogens (co-detections).

In Scotland, community transmission of *M. pneumoniae* is monitored by a sentinel respiratory surveillance system, with general practitioners (GPs) collecting upper respiratory tract swabs for microbiological testing from patients presenting with acute respiratory infection (ARI) [29]. The programme has been effective in its current format since 2022, when the World Health Organization developed new guidance for monitoring syndromic ARI [30]. Expected seasonal patterns are detected, with appearance of respiratory syncytial virus (RSV) particularly in older (≥ 65 years) and younger (˂ 4 years) age groups through autumn and winter, as well as with occurrence of influenza virus affecting all age groups in winter, and with notable SARS-CoV-2 activity in summer periods of 2022/23 and 2023/24 in older age groups. These three pathogens are the main drivers of simultaneous increases in hospitalisation rates for ARI in Scotland, although increased hospital admissions for other common viral respiratory pathogens are also observed in winter seasons [31].

Here we present a detailed report on the epidemiology of *M. pneumoniae* ARIs detected in primary care in Scotland since the implementation of the sentinel surveillance system, from International Organization for Standardization (ISO) week 40 2022 to the end of ISO week 20 2025. We also report on co-detection with nine common viral respiratory pathogens and the numbers of *M. pneumoniae* associated hospital admissions.

## Methods

### Community Acute Respiratory Infection sentinel surveillance

The Community Acute Respiratory Infection (CARI) sentinel surveillance programme was established in Scotland in October 2022, as national community testing programmes for SARS-CoV-2 were discontinued. The programme, supported by the Scottish Government and operated by Public Health Scotland, has a network of over 100 representative sentinel GP sites across Scotland (Laird, 2025). The GPs recruit and take nasopharyngeal swabs from a convenience sample of around 30% of patients presenting with symptoms of ARI. All swabs are tested for 10 respiratory pathogens, using an in-house developed multiplex PCR. The 10 pathogens are *M. pneumoniae*, influenza A virus, influenza B virus, SARS-CoV-2, RSV, adenovirus, rhinovirus, seasonal coronavirus, parainfluenza virus and human metapneumovirus.

In Scotland, a unique patient identifier, known as the Community Health Index number, is allocated to all patients when they register with a GP. This enables record-linkage of data collected through the CARI programme to be linked with other National Health Service Scotland datasets, including the Scottish Morbidity Record of inpatient activity (SMR01). This is a clinically validated dataset of all inpatient admissions, that has international classification of diseases (ICD)10 diagnosis codes added retrospectively and was used to identify hospital admissions in *M. pneumoniae* cases.

### Test positivity for* Mycoplasma pneumoniae* by sociodemographic characteristics

Data from the CARI system were used to describe the epidemiology of *M. pneumoniae* from ISO week 40 2022 to the end of ISO week 20 2025, covering three winter seasons (2022/23, 2023/24 and 2024/25). The total number of tests and the number that were positive for *M. pneumoniae,* regardless of whether there was another pathogen detected, were derived by week across the study period. To provide a picture of the epidemiology and burden of *M. pneumoniae*, the proportion of positive tests (test positivity) was stratified by age, sex, and Scottish index of multiple deprivation quintile (SIMD). There were three variables for sex (male/female/unknown) and SIMD quintile is a postcode-linked indicator of relative deprivation measured across income, employment, education, health, access to services, crime and housing [32].

### Co-detections of* Mycoplasma pneumoniae *and other respiratory pathogens

For samples that had been successfully tested for every pathogen (i.e. complete tests only; any sample that returned an invalid PCR result for an individual pathogen in the multiplex panel being omitted) we determined the number of samples that returned simultaneous PCR positive results for more than one pathogen and reported them as co-detections. Proportions of co-detections by age groups were compared using chi-square tests. Of the total of *M. pneumoniae* co-detections, we calculated the proportion represented by each individual viral pathogen, stratified by age. The degree to which these proportions reflect the incidence of pathogens occurring in the test population is indicated by the ratio of number of detections of a given pathogen vs number of detections of the same pathogen in co-detection with *M. pneumoniae* in the CARI population.

### Epidemiological assessment of patients with severe disease

To assess severity, SMR01 was searched for patients in the CARI population with complete tests (as defined above) who were admitted to hospital as an emergency within 14 days of the positive test, and who had a respiratory ICD10 diagnosis code in the primary field. Patients were stratified by sex, age, SIMD quintile and whether a co-detection was present.

### Statistical analyses

Differences between age, sex and SIMD quintile groups in test positivity and co-detection proportions, and differences in ratios of single vs co-detections of pathogens, were all assessed using chi-square tests of equal proportions. Tests with samples < 5 were confirmed using Fisher’s exact test.

## Results

### Test positivity

**Figure 1:**
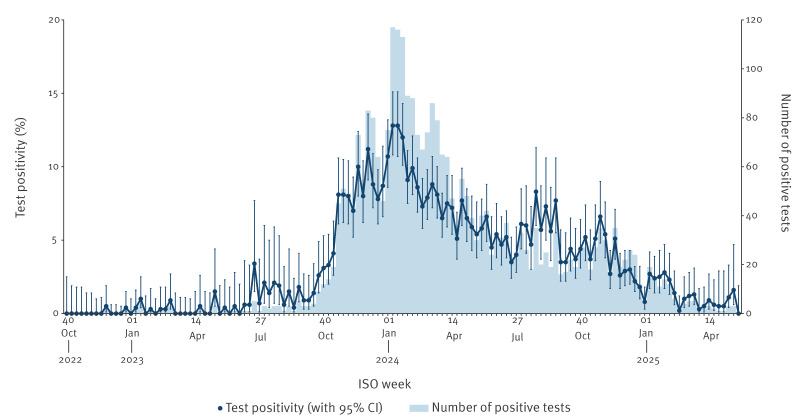
Number of positive tests and test positivity for *Mycoplasma pneumoniae* in the CARI programme, Scotland, October 2022–May 2025 (n = 65,798 tests)

CARI: Community Acute Respiratory Infection; CI: confidence interval; ISO: International Organization for Standardization.

Over the study period, 65,798 viable swabs were taken from patients recruited to the CARI programme, including 40,158 female, 25,534 male and 106 individuals with no data on sex; overall, the patients’ median age was 35 years (interquartile range (IQR): 17–59), and 35,890 (54.5%) tested positive for any pathogen. A total of 3,031 swabs (4.6%, 3,031/65,798) tested positive for *M. pneumoniae* (from 1,686 female, 1,340 male and five with sex unknown patients; median age: 18 years, IQR: 9–39) of which a subset of 2,799 (92.3%) swabs had complete test results for all pathogens. Figure 1 shows the number of tests positive *for* *M. pneumoniae* over time, alongside test positivity for the study period.

The number of *M. pneumoniae* positive tests returned weekly increased from baseline numbers from week 39 2023 and reached a peak of 117 in week 2 2024. Test positivity was very low in the community across the 2022/23 season, with test positivity reaching a maximum in June 2023 (ISO week 26) at 3.4% (5/147). Values began to rise in 2023/24, peaking at 12.8% (117/915) in January (ISO week 2) and remaining high for the remainder of the season. While test positivity generally declined through the 2024/25 season and did not reach the 2023/24 peak, *M. pneumoniae* remained present in the community.

**Figure 2:**
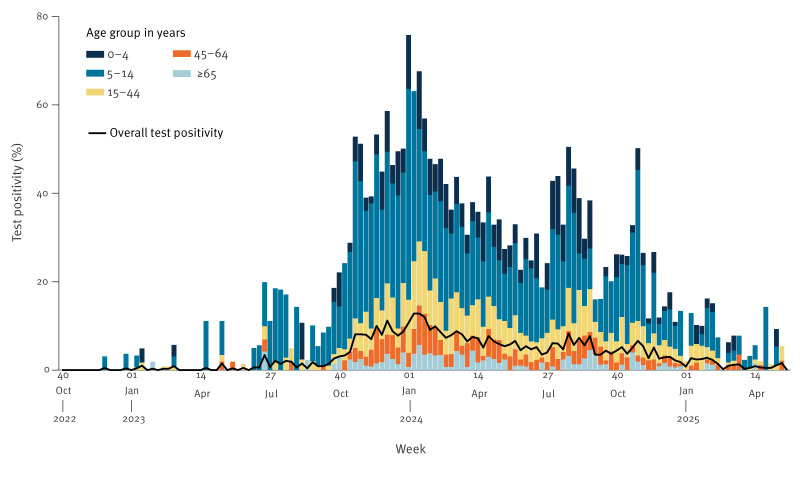
Test positivity for *Mycoplasma pneumoniae* in the CARI programme by age group, Scotland, October 2022–May 2025 (n = 65,798 tests)

CARI: Community Acute Respiratory infection; ISO: International Organization for Standardization.

*M. pneumoniae* disproportionately affected the age group of 5–14 years, for which test positivity peaked at 47.7% (31/65) in ISO week 1, 2024 (Figure 2). In 2022/23, there was a statistically significant difference in test positivity between the 5–14-year age group and all other age groups (p < 0.05). There were statistically significant differences in test positivity between all age groups in 2023/24 (p < 0.05), but no differences between any of the age groups in 2024/25. There was a statistically significant difference in test positivity between females and males in season 2022/23 and 2023/24 (p < 0.05) but not in season 2024/25. There were no statistically significant differences in SIMD for the two seasons for which data were available.

Overall *M. pneumoniae* test positivity for the season was higher in 2023/24 for every age group and was highest in 5–14-year-olds at 20.1% (956/4,761) (Table 1). Test positivity was only marginally lower for females each year, and there was no indication of change in test positivity related to SIMD.

**Table 1:** *Mycoplasma pneumoniae* test positivity for each season, stratified by sex, age and SIMD quintile, Scotland, 2022/23*–*2024/25 (n = 65,798 tests)

Season	2022/23			2023/24			2024/25 until week 20		
	Positive samples	Samples tested	Test positivity % (95% CI)	Positive samples	Samples tested	Test positivity % (95% CI)	Positive samples	Samples tested	Test positivity % (95% CI)
**Overall**									
Overall	70	14,925	0.5 (0.4–0.6)	2,530	34,243	7.4 (7.1–7.7)	431	16,630	2.6 (2.4–2.8)
**By sex**									
Female	35	9,330	0.4 (0.3–0.5)^*^	1,398	20,746	6.7 (6.4–7.1)^*^	253	10,082	2.5 (2.2–2.8)
Male	35	5,587	0.6 (0.5–0.9)^*^	1,128	13,424	8.4 (7.9–8.9)^*^	177	6,523	2.7 (2.3–3.1)
Unknown	0	8	0.0 (0.0–32.4)	4	73	5.5 (2.2–13.3)	1	25	4.0 (0.7–19.5)
**By age group in years**									
0–4	4	1,601	0.2 (0.1–0.6)	180	2,919	6.2 (5.4–7.1)^**^	31	1,630	1.9 (1.3–2.7)
5–14	32	1,852	1.7 (1.2–2.4)^**^	956	4,761	20.1 (19.0–21.2)^**^	135	1,729	7.8 (6.6–9.2)
15–44	25	5,518	0.5 (0.3–0.7)	931	12,053	7.7 (7.3–8.2)^**^	197	6,198	3.2 (2.8–3.6)
45–64	5	3,416	0.1 (0.1–0.3)	333	8,397	4.0 (3.6–4.4)^**^	51	4,210	1.2 (0.9–1.6)
≥ 65	4	2,531	0.2 (0.1–0.4)	127	6,096	2.1 (1.8–2.5)^**^	17	2,856	0.6 (0.4–1.0)
Unknown	0	7	0.0 (0.0–35.4)	3	17	17.6 (6.2–41.0)	0	7	0.0 (0.0–35.4)
**By SIMD**									
1 (most deprived)	Data not available			464	6,216	7.5 (6.8–8.1)	72	3,433	2.1 (1.7–2.6)
2				425	5,116	8.3 (7.6–9.1)	69	2,892	2.4 (2.0–3.0)
3				358	4,452	8.0 (7.3–8.9)	64	2,665	2.4 (1.9–3.1)
4				417	4,610	9.0 (8.3–9.9)	49	2,475	2.0 (1.5–32.6)
5 (least deprived)				382	4,543	8.4 (7.6–9.3)	67	2,450	2.7 (2.2–3.5)
Unknown				484	9,306	5.2 (4.8–5.7)	110	2,715	4.1 (3.4–4.9)

CI: confidence interval; SIMD: Scottish Index of Multiple Deprivation.

^*^ p < 0.05 compared to other sex category.

^**^ p < 0.05 compared to all other age groups.

Overall, 1.8% (55/3,031) of patients positive for *M. pneumoniae* were admitted to hospital within 14 days of the CARI test. These patients all had a respiratory diagnosis code as the primary cause of admission.

### *Mycoplasma pneumoniae* and respiratory virus co-detection

Of 2,799 positive *M. pneumoniae* samples that returned results for all nine other pathogens, 728 had a positive PCR result for one other pathogen or more (26.0% co-detection positivity). For the other pathogens in our panel, co-detection proportions for each ranged from 16.0% (299/1,867) for influenza B virus to 48.6% (839/1,725) for adenovirus.

**Figure 3:**
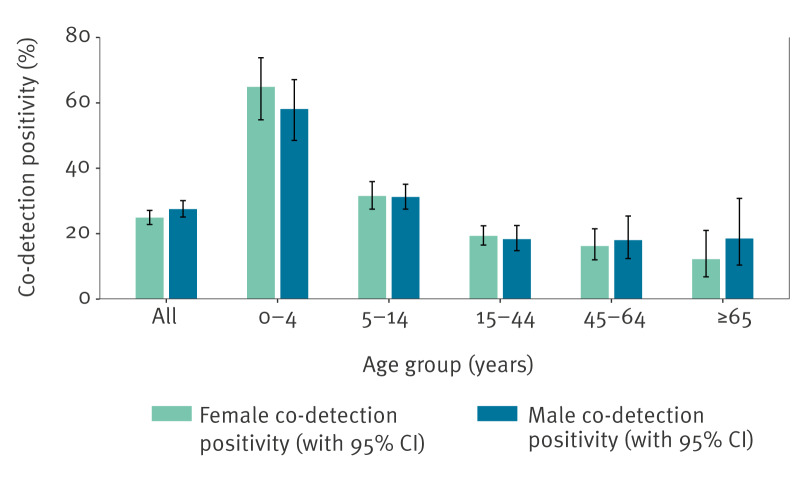
Co-detection test positivity involving *Mycoplasma pneumoniae* and any other pathogen in the CARI programme, by age and sex, Scotland, October 2022–May 2025 (n = 2,799 complete tests)

CARI: Community Acute Respiratory Infection; CI: confidence interval.

Of the 728 *M. pneumoniae* co-detections, 87.8% (639/728) were with one other pathogen only, 11.3% (82/728) with a maximum of two others and 1.0% (7/728) with three or more other pathogens. Co-detections were found in all age groups but substantially more by proportion in the 0–4-year age group. Of 199 *M. pneumoniae* positive test results, 122 in this youngest age group had simultaneous detection of other pathogens (61.3% co-detection positivity), and there was co-detection in 321 of 1,024 cases aged 5–14 years (31.3% co-detection positivity) (Figure 3). Co-detection positivity continued to decline as age group increased with statistically significant differences between the 0–4-year age group and all others (chi-square test p <0.0001 for all pairwise comparisons), and the 5–14-year age group and all others (p <0.0001 for all pairwise comparisons). There were no statistically significant differences in co-detection positivity between males and females overall (p = 0.1253) nor SIMD (p *=* 0.3468)*.*

**Figure 4:**
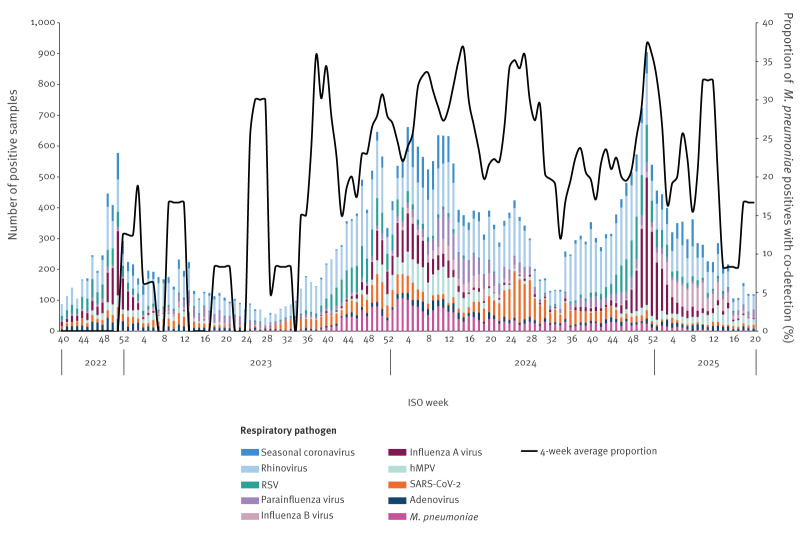
Number of positive samples of respiratory pathogens and 4-weekly average proportion of *Mycoplasma pneumoniae* cases with occurrence of co-detection in the CARI programme, Scotland, October 2022–May 2025 (n = 65,798 complete tests)

CARI: Community Acute Respiratory Infection; hMPV: human metapneumovirus; ISO: International Organisation for Standardisation; RSV: respiratory syncytial virus.

Across the study period, rhinovirus circulation was present throughout each year, but the number of weekly detections of some of the other respiratory pathogens varied with a clear seasonal pattern. Influenza viruses, RSV, seasonal coronavirus and hMPV occurred more frequently in autumn and winter (approximately weeks 40 through 8, each year). In contrast, parainfluenza viruses were frequently observed in spring (around weeks 10 to 24) and, for 2024, in autumn (weeks 36 to 50). While SARS-CoV-2 did not have an established seasonal pattern, a period of elevated incidence during the summer of 2024 could be seen (roughly weeks 20 to 40). In this study, *M. pneumoniae* activity was high from week 39 2023 through to the last weeks of 2024. During this time, fluctuation in the average proportion of *M. pneumoniae* cases which were simultaneously affected by other virus(es) followed, for the most part, seasonal increases and decreases in the overall number of positive respiratory tests and was not obviously attributable to the occurrence of any individual virus (Figure 4). 

**Figure 5:**
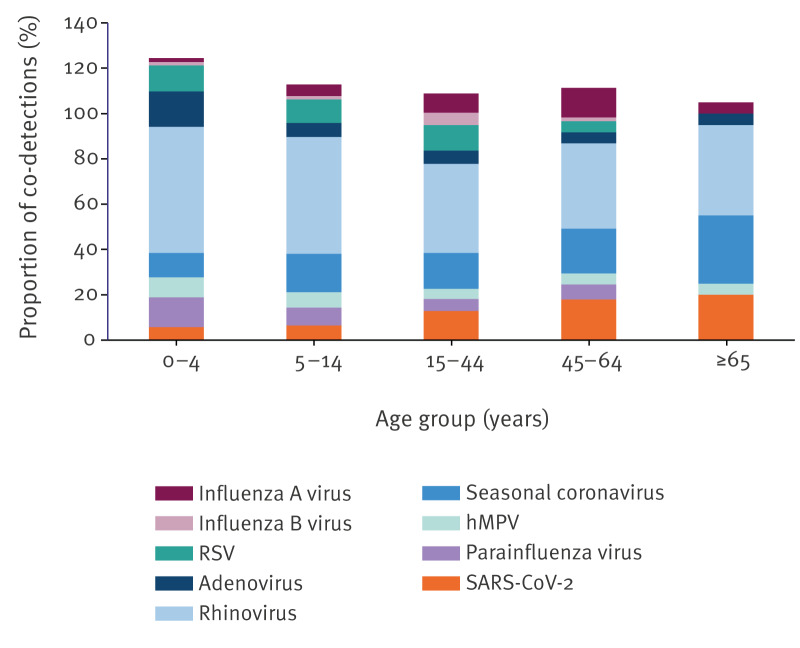
Proportion of *Mycoplasma pneumoniae* co-detections by pathogen in the CARI programme, by age, Scotland, October 2022–May 2025 (n = 728 co-detections)

CARI: Community Acute Respiratory Infection; hMPV: human metapneumovirus; RSV: respiratory syncytial virus.

Across all ages, rhinovirus was the most frequently co-detected with *M. pneumoniae* by proportion (346/728; 47.5%; 95% CI: 43.9–51.2), with the highest proportion in the 0–4-year age group where it accounted for 68/122 (55.7%; 95% CI: 46.9–64.2) of *M. pneumoniae* co-detections (Figure 5). Adenovirus was the next most frequently identified in the 0*–*4-year age group with 19/122 of co-detections (15.6%; 95% CI: 10.2–23.0). Other viruses of childhood, parainfluenza virus and RSV, were also detected at higher proportions in *M. pneumoniae* co-infections in children than adults. Increased proportions of SARS-CoV-2, seasonal coronavirus (non-SARS-CoV-2) and influenza A virus were co-detected with *M. pneumoniae* in most adult age groups (Figure 5).

On the Y axis of the graph, totals greater than 100% are due to co-detection with one or more other pathogens.

The frequency of any pathogen being detected alone vs being co-detected with *M. pneumoniae* is shown as a ratio in Table 2. The highest frequency of detection overall was with seasonal coronavirus, with one co-detection occurring for every 28.6 detections of seasonal coronavirus alone. This was followed by adenovirus for which one co-detection happened for every 30.4 single detections of this virus. The lowest frequency of co-detection overall was with influenza A virus (one co-detection occurring for every 100.5 detections of influenza A virus alone). Relative to circulating levels in the community, the frequency of co-detection was highest for SARS-CoV-2 in the 0–4-year age group (one per 28.7 detections of SARS-CoV-2). On the other hand, in the 5–14-year age group the frequencies of co-detections with seasonal coronavirus and SARS-CoV-2 were most elevated, with one occurrence of co-detection of seasonal coronavirus per 8.4 single detections of this virus and one co-detection of SARS-CoV-2 per 11.1 detections of SARS-CoV-2 alone. In the 15–44-year age group, co-detection comprising RSV was most frequent (one per 30.3 detections of RSV) and in the 45–64-year age group co-detections including adenovirus (one per 50.7 detections of adenovirus). Numbers of co-detections in the ≥ 65-year age group were generally low, and this measure was less reliable.

**Table 2:** Numbers of positive test results and *Mycoplasma pneumoniae *co-detection results by age group and by pathogen, as well as the ratio of *M. pneumoniae* co-detection, Scotland, October 2022–May 2025 (n = 65,798 complete tests)

Pathogen^a^	All ages (n = 65,798)			0–4 years (n = 6,150)			5–14 years (n = 8,342)			15–44 years (n = 23,769)			45–64 years (n = 16,023)			≥ 65 years (n = 11,483)			Age unknown (n = 31)		
	*M. pneumoniae* co-detection^a^		Ratio	*M. pneumoniae* co-detection^a^		Ratio	*M. pneumoniae* co-detection^a^		Ratio	*M. pneumoniae *co-detection^a^		Ratio	*M. pneumoniae* co-detection^a^		Ratio	*M. pneumoniae* co-detection^a^		Ratio	*M. * *pneumoniae* detection^a^		Ratio
	+	-		+	-		+	-		+	-		+	-		+	-		+	-	
**Influenza A virus**	44	4,420	100.5^b,c,d,e,f^	2	309	154.5	16	458	28.6	17	1,773	104.3^b,e^	8	1,329	166.1	1	548	548	0	3	3/0
**Influenza B virus**	19	1,848	97.3^c,d,e^	2	85	42.5	5	191	38.2	11	1,309	119^b,e^	1	212	212	0	48	48/0	0	3	3/0
**RSV**	73	3,229	44.2^g^	14	845	60.4	33	407	12.3	23	696	30.3^g,h^	3	735	245	0	543	543/0	0	3	3/0
**Adenovirus**	55	1,670	30.4^g,h,i,j^	19	675	35.5	20	303	15.2	12	472	39.3	3	152	50.7	1	68	68	0	0	0/0
**Rhinovirus**	346	12,423	35.9^g,h,j^	68	2,157	31.7	166	1,941	11.7	80	4,122	51.5	23	2,368	103	8	1,829	228.6	1	6	6
**Seasonal coronavirus**	117	3,352	28.6^f,g,h,i,j^	13	482	37.1	54	456	8.4	32	1,115	34.8^g,h^	12	775	64.6	6	522	87	0	2	2/0
**hMPV**	46	2,653	57.7^c,e^	11	444	40.4	22	351	16	9	688	76.4	3	642	214	1	525	525	0	3	3/0
**Parainfluenza virus**	56	2,848	50.9^g,e^	16	580	36.3	25	370	14.8	11	735	66.8	4	639	159.8	0	523	523/0	0	1	1/0
**SARS-CoV-2**	69	4,000	58^c,d,e^	7	201	28.7	21	234	11.1	26	1,376	52.9	11	1,210	110	4	979	244.8	0	0	0/0
***M. pneumoniae^k^***	2,071	NA	NA	77	NA	NA	703	NA	NA	870	NA	NA	301	NA	NA	118	NA	NA	2	NA	NA

hMPV: human metapneumovirus; NA: not applicable; RSV: respiratory syncytial virus.

^a^ *M. pneumoniae* co-detections are the joint detection of *M. pneumoniae* and the pathogen indicated in the row heading (+), while (–) denotes detection of the pathogen in the row heading in the absence of *M. pneumoniae*.

^b^ Compared with RSV (p < 0.05).

^c^ Compared with adenovirus (p < 0.05).

^d^ Compared with rhinovirus (p < 0.05).

^e^ Compared with seasonal coronavirus (p < 0.05).

^f^ Compared with parainfluenza virus (p < 0.05).

^g^ Compared with influenza A virus (p < 0.05).

^h^ Compared with influenza B virus (p < 0.05).

^i^ Compared with hMPV (p < 0.05).

^j ^Compared with SARS-CoV-2 (p < 0.05).

^k^ This row is for single detections of *M. pneumoniae*.

Except for the first column heading, the main column headings of the table display the number of tests conducted either overall or per age group. Numbers inside the column, however, refer only to tests that turned out positive for one pathogen or more, so the totals in main columns are less than the number in the column heading. For patients with testing completed for all pathogens, the proportion admitted to hospital was 1.5% (11/728) for patients when *M. pneumoniae* was co-detected with another pathogen, and 1.9% (40/2,071) when detected alone. This was not a statistically significant difference (p *=* 0.466).

Overall, 11 (1.5%) of the 728 patients where *M. pneumoniae* was co-detected with another pathogen were admitted to hospital within 14 days of the CARI test.

## Discussion

The last notable epidemic of *M. pneumoniae* in the United Kingdom (UK) and Europe occurred in the 2019 to 2020 winter period [22]. A previously described pattern in the UK has been epidemics every 4 years, mostly affecting school aged children, with low-level sporadic infection in between [33]. In Scotland, the CARI surveillance system has closely monitored *M. pneumoniae* infection since 2022. The data suggest that low-level infection was suppressed by non-pharmaceutical interventions during the COVID-19 pandemic, with its continued global suppression through 2021/22 generating speculation that *M. pneumoniae* might even be eradicated [18]. However, in Scotland in the autumn of 2023, a resurgence of *M. pneumoniae* occurred, with similar events also reported from other countries in the northern hemisphere at the same time. This relatively late re-emergence compared with other pathogens is thought to be attributable in part to the slow generation interval of *M. pneumoniae* [7].

During a 6 month-period in winter 2023/24 there were 2,313 detections of *M. pneumoniae* in the CARI surveillance system. While this appears to be a relatively large wave of infection, it is difficult to ascertain as comparative data are lacking for the pre-pandemic period; surveillance of *M. pneumoniae* prior to COVID-19 relied mainly upon a small number of laboratory detections of samples collected during routine clinical practice, either in primary care by GPs or in hospitals. Since then, the CARI surveillance system has captured an estimated 7.3% of all acute respiratory consultations in Scotland from October to March 2023/24. This provides a robust baseline of post-pandemic data to benchmark future community surveillance of *M. pneumoniae.*

The CARI surveillance system also demonstrated that a quarter of detections of *M. pneumoniae* were co-detected with other viral respiratory pathogens. This proportion was higher in younger age groups than older, with 122/199 (61%) in the 0–4-year age group and 321/1,024 (31%) in the 5–14-year age group. This is in the range of values globally reported across various studies of hospitalised paediatric MPP [8-10,24-28] and suggests that *M. pneumoniae* may co-infect children with respiratory pathogens at a level in the community, that is similar to that of cases managed in secondary care. In the CARI system, and as a commonly held observation, there was a higher overall test positivity and higher diversity of respiratory pathogens affecting children than seen for adults at any given time period [34], likely to be attributable to many factors including the relatively naive immune status of children and the ease of transmission of respiratory pathogens in settings where children are in close proximity with each other for extended periods. For this reason, it is perhaps not surprising that the level of co-detected pathogens was found to be higher in this age group too.

The proportions of co-detections of *M. pneumoniae* with specific pathogens show some variance between age groups, with younger ages having a higher proportion of co-detections with viruses common to childhood, and proportions of co-detection with SARS-CoV-2, seasonal coronavirus and influenza all increasing with age. To account for background prevalence of viruses in the population we looked at the ratio (likelihood) of single virus detection vs co-detection with *M. pneumoniae* in each age group*.* We found some indication that particular co-detections were more likely than others; overall, co-detection was most likely with seasonal coronavirus and adenovirus, and least likely with influenza. Within the 5*–*14-year age group specifically, who were most affected by the *M. pneumoniae* epidemic by our measures, seasonal coronavirus and SARS-CoV-2 were most likely to be co-detected with *M. pneumoniae* with co-detection occurring for every eight detections of seasonal coronavirus, and every 11 of SARS-CoV-2. We were not able to determine if these co-detections were occurring simultaneously or sequentially, but underlying mechanisms such as competition for receptors, cellular resources and host-mediated immune responses affect interactions of respiratory pathogens, determining infection outcomes [35,36]. Co-detections resulting from positive interactions between pathogens could explain, at an individual level, the associations we observed where synergism might be occurring. Future work on the dynamics of co-prevalence could further help understanding of co-detections at population level [37,38].

Several limitations to the interpretation of surveillance data shared in this study are worth noting. All the described incidences of co-detection of *M. pneumoniae* with viral respiratory pathogens have been in patients attending GPs with symptoms of ARI. Hospital admission proportions were similar between patients with single and co-detection of *M. pneumoniae*, but the overall number of patients with *M. pneumoniae* who were subsequently admitted to hospital is very low and larger sample sizes would be needed to reliably assess this difference. It is also possible that more serious cases of *M. pneumoniae* infection (including cases in children) will bypass consultation in the community and be admitted to hospital directly, and that community cases identified through CARI surveillance are less severe.

We were unable to determine whether *M. pneumoniae* was the cause of disease in patients, and children can be asymptomatic carriers [39-41]. Without knowing whether PCR signals were produced from actively replicating virus or persistence of target nucleic acid, we have purposefully used the term co-detection rather than co-infection, and we do not provide relative strengths of PCR signals from co-detection samples. Neither have we distinguished between related virus strains within positive test results, for example parainfluenza types 1*–*4. Inherent bias in the multiplex PCR methods used should also be considered; amplification of multiple targets in samples may be unequal in some circumstances. Lastly, vaccination status of individuals was not considered. Seasonal programmes to vaccinate risk groups against influenza, RSV and COVID-19 are delivered annually in Scotland. Likelihood of detection and severity of disease outcomes (hospital admission) will both be affected by vaccine effectiveness, and by implication underlying mechanistic pathways of pathogen interactions (antagonistic or synergistic) affecting co-detection.

## Conclusion

Sentinel surveillance of respiratory pathogens in the community was helpful to characterise an *M. pneumoniae *epidemic, revealing frequent respiratory-pathogen co-detections in children under 14-year-olds, as reported in hospitalised *M. pneumoniae* paediatric cases. Co-detections of *M. pneumoniae* in symptomatic children in the community could be a useful indicator of co-infecting incidence in admitted cases of more severe acute illness and trigger wider awareness and clinical testing for *M. pneumoniae* when community incidence is high. Early detection of potentially more complex clinical cases could enable targeted case management, including specific antibiotic prescribing and sequence analysis where macrolide resistance is a concern. As a significant pathogen of childhood respiratory illness with known seasonal epidemic occurrence, it is important that surveillance of *M. pneumoniae* and co-detection incidence in children continues to be closely monitored.

## Data Availability

Public Health Scotland Open Data is available at Viral Respiratory Diseases (Including Influenza and COVID-19) Data in Scotland - Datasets - Scottish Health and Social Care Open Data.
